# Importance of surface topography on pulsed laser-induced damage threshold of Sapphire crystals

**DOI:** 10.1038/s41598-017-01192-7

**Published:** 2017-04-28

**Authors:** Benoît Bussière, Nicolas Sanner, Marc Sentis, Olivier Utéza

**Affiliations:** 1 Aix Marseille Université, CNRS, LP3 UMR 7341, 13288 Marseille, France; 2 Amplitude Technologies, CE2926, 91029 Evry cedex, Lisses, France

## Abstract

We measure the laser-induced damage threshold (LIDT) fluence under single shot at the surface of Sapphire samples prepared following the standards of two methods yielding to different surface finish and used in optical and laser industry. We use AFM microscopy to measure the roughness parameter Ra and power spectral density (PSD) of the sample surface. We show that the quality of surface topography resulting from surface preparation affects the damage threshold of Sapphire crystals exposed to femtosecond, picosecond, and nanosecond laser conditions at visible and near-infrared wavelengths. We observe a higher resistance to laser damage or macroscopic modification when the surface finish presents a smooth and regular topography. We indeed measure a 1.4 to 2 times increase of the LIDT fluence in femtosecond and picosecond regimes and up to 5 times with nanosecond pulses. Using simple damage model and PSD data, we correlate the LIDT reduction of Sapphire samples of lower quality of surface finish with the high-frequency tail component of their PSD distribution corresponding to striations of the width of a fraction of the laser wavelength. This study emphasizes the importance of detailed assessment of surface topography for laser damage evaluation and understanding and for indicating directions of improvement.

## Introduction

Due to its hardness and transparency in visible, UV and mid-IR and its thermal stability, biocompatibility and high resistance to corrosion, Sapphire is a material of high interest in optics, photonics, and laser technology^1^. For instance, Sapphire is widely used as optical windows, waveplates or substrates for complex optical devices and it can be also interesting to machine in order to develop advanced optical components^2–4^. Moreover, when doped, for instance with Titanium ions, it forms Ti:Sapphire active material which is a key laser material in the context of the development of high-peak-power laser systems^5^. The evaluation of the laser-induced damage threshold (LIDT) fluence at the surface of Sapphire is important for its successful integration and reliable use in optical, laser or photonics systems^6–8^. As Sapphire is extremely difficult to machine making delicate the realization of high-quality optical devices, one will also find interest in the accurate determination of the fluence levels required to modify the surface of Sapphire under a wide range of laser exposure conditions. Otherwise, the last step of fabrication of an optical component, consisting in the preparation of the material surface to provide the optics with optimal optical properties, is essential^9–11^. Indeed, the initial conditions of the surface, such as the roughness, topography and chemical and geometrical structure (contaminants, lattice deformation, inclusions), may have an influence on the absorption of the laser light, acting as absorption precursor or enhancement factor. As a consequence, a different response of the material to laser damage or to laser ablation and surface modification can be observed.

In this work, we focus on the influence of surface topography on LIDT and more precisely on the resistance to laser-induced modification of the surface of crystalline Sapphire (Al_2_O_3_) samples prepared with two different methods leading to the different surface finish. The first one (referred as type A method) is the classical technique of surface preparation used in the optics industry to provide high-quality Sapphire optical components. The second one (type B method) is the procedure to prepare Titanium-doped Sapphire (Ti:Al_2_O_3_) crystals for high peak power femtosecond laser systems. Indeed, the surface finish is different in this last case, since the doping prevents to obtain the same quality of the surface. Even if the process of surface finish may promote additional pollution of the sample and also modify its sub-surface structure^9, 10, 12–16^, we focus here our study on the particular influence of the characteristics of surface topography on the energy level requested for macroscopic surface modification. We first use Atomic Force Microscopy (AFM) for detailed surface characterization and we further expose the samples to femtosecond, picosecond, and nanosecond laser illumination conditions in single pulse regime at visible and near-infrared wavelengths. We finally establish a correlation between the characteristics of the surface topography and the evolution of LIDT fluence in different regimes of pulse duration (ns to fs). Our results demonstrate the importance of measuring the power spectral density (PSD) of the surface of the samples for understanding the damage response of the studied samples exposed to pulsed irradiation. Furthermore, we extend our measurements to the wavelength of 1030 nm corresponding to commercial femtosecond laser systems used in industry. We thus provide an original set of LIDT fluence data obtained on several decades of pulse duration and under different laser wavelengths using the same evaluation protocol. Those measurements complement former LIDT measurements and laser damage and structuration databases, already available in the literature for amorphous and crystalline Sapphire from femtosecond to millisecond pulse duration^6, 17–20^.

## Results

### Details of surface topography of the samples

**Figure 1:**
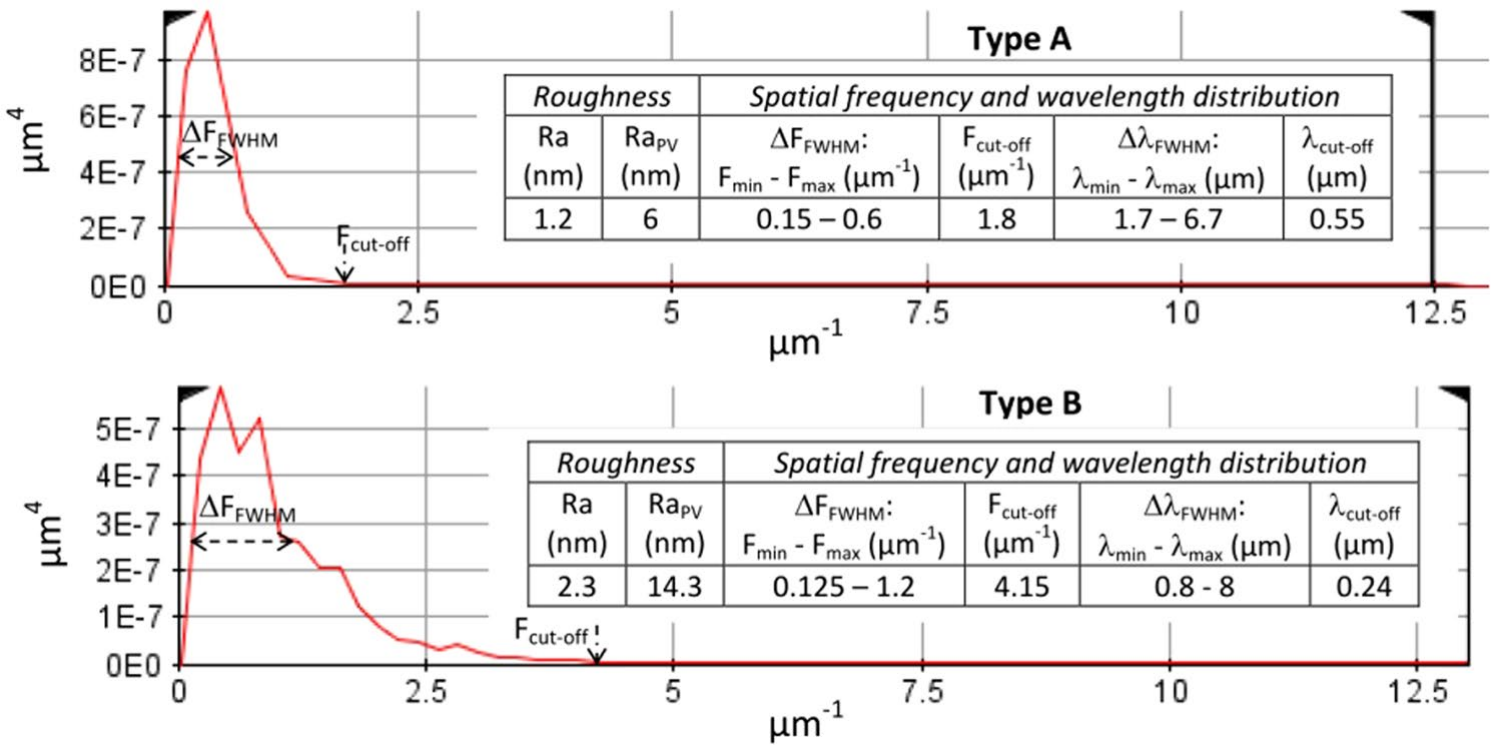
Surface topography of samples. Power spectral density of the surface vs spatial frequency for two Sapphire samples prepared with type A (top) and type B (bottom) methods. The tables in the insets show the parameters extracted from the PSD distribution and characterizing the surface topography of the samples. The Ra parameter is the roughness average and Ra_PV_ the peak-to-valley roughness. ΔF_FWHM_ (F_min_ − F_max_) and Δλ_FWHM_ (λ_min_ − λ_max_) respectively correspond to the extent (measured at FWHM to the peak) of the spatial frequency and wavelength bandwidth of PSD distribution of the tested surfaces. F_cut-off_ (λ_cut-off_) informs on the high-frequency tail of the PSD distribution, specifying the highest spatial frequency (lowest spatial wavelength) present on the surface.

Quantitative parameters describing the surface topography of the sample measured by an AFM microscopy system (PSIA XE-100) are summarized in Fig. 1.

Basically, Sapphire samples prepared following type B procedure show a high roughness with a high Ra and an extended PSD distribution with high-frequency components. On the other hand, type A method for surface preparation yields a smoother surface topography free of high-frequency modulations. Even if type B surface finish of the material appears not be optimal for Sapphire according to the details of its surface topography, the roughness is however kept sufficiently low to minimize the losses by scattering and provide optics of good quality. For instance, considering reflectivity, the losses can be estimated by the equation, ${{\boldsymbol{R}}}_{{\boldsymbol{scatter}}}/{{\boldsymbol{R}}}_{{\boldsymbol{i}}}\approx {\bf{e}}{\bf{x}}{\bf{p}}[{(\frac{-4{\boldsymbol{\pi }}Ra}{{\boldsymbol{\lambda }}})}^{2}]$, ref. 21 giving the reflectivity R_scatter_ scattered by a rough surface (with Ra characteristics) as compared to a perfectly smooth surface R_i_. In the worst case (type B), the proportion of reflected light scattered by the surface (<1%) remains negligible.

### Damage evaluation

To get LIDT data under different pulse exposure (pulse duration, wavelength) but that can be easily compared, we performed the damage tests in operating conditions as constant as we could achieve. In particular, we realized the tests with a nearly constant beam size (w_0_ ~ 10 µm) corresponding to a scale that is often used for surface micro-structuration. The motivation was also to measure the surface damage (and not the one of the bulk) and to avoid any measurement skew related to the use i) of large beam size, which would imply higher sensitivity of the beam to nonlinear propagation effects (especially considering the femtosecond laser beam), and ii) of different beam sizes. Indeed, considering this latter case, LIDT fluence may vary (be reduced when increasing beam size) depending on the eventuality to intercept highly absorbing defects of low spatial density^22, 23^. This is especially relevant in the case of nanosecond laser excitation for which high sensitivity of the damage phenomenon to the presence of defects has been reported^24–26^. For femtosecond pulses, the sensitivity to the defects is reduced due to the higher intensity used, which favors nonlinear absorption on the electrons of the valence band. As a result, the damage tends to be deterministic^26, 27^. So, as a general comment on the damage probability curves and LIDT values that are retrieved, the constancy of our experimental conditions (except obviously the pulse duration and wavelength) makes possible the comparison of the damage data. This allows us to study the influence of the surface topography resulting from surface preparation on the damage behavior of Sapphire crystals exposed to an extended range of laser exposures (fs to ps, infrared and visible).

**Figure 2:**
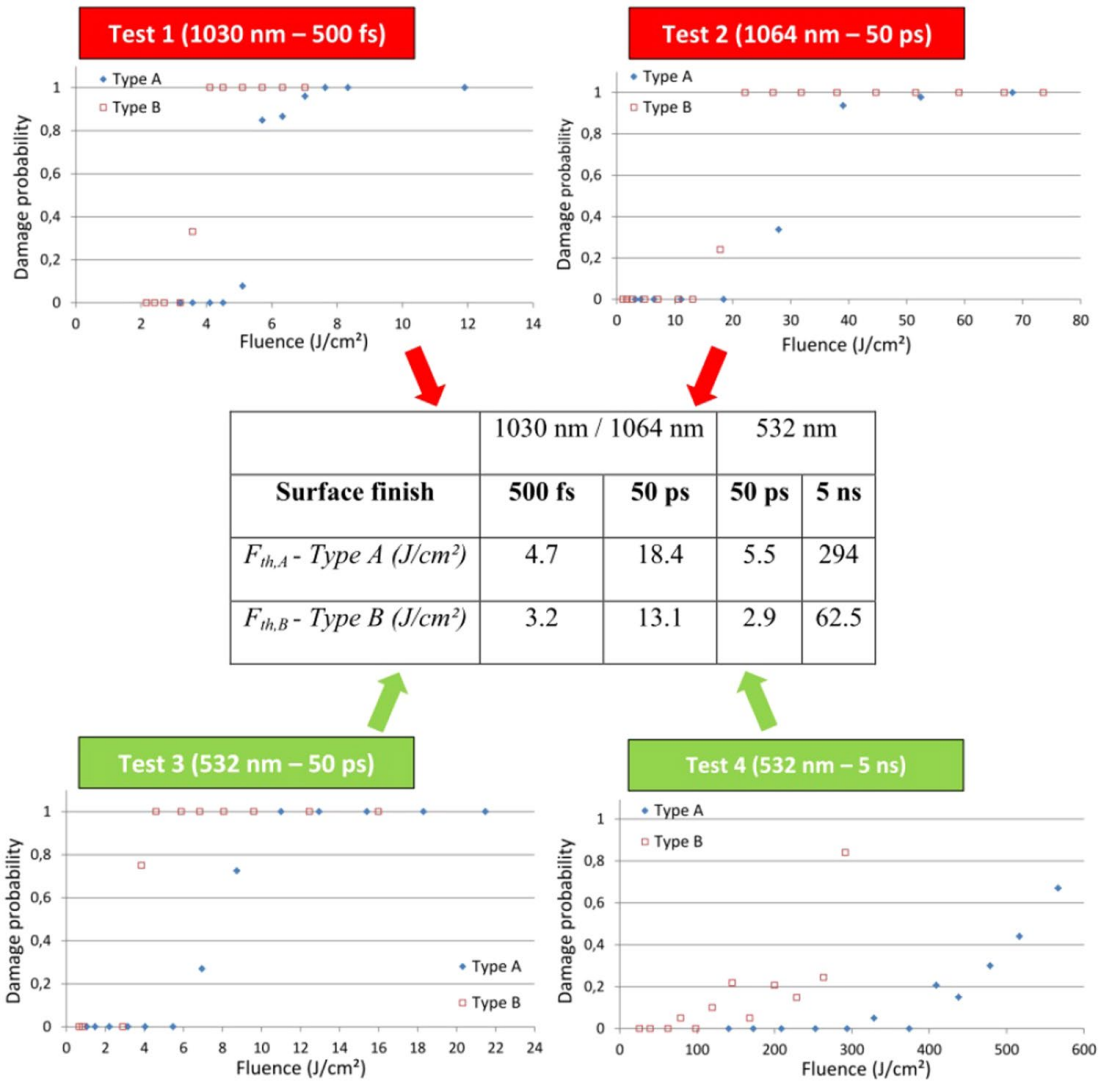
LIDT Measurements. Damage probability curves under various irradiation conditions and in single shot regime obtained for two kinds of Sapphire samples only differing by their surface topography as a result of the different procedures of surface preparation. Each point is averaged on 20 independent tests (error bar not shown). The LIDT fluences are recalled in the table in the central inset.

We performed four irradiation tests on “type A” and “type B” Sapphire samples in air under normal incidence and in the single shot regime with different pulse durations (500 fs, 50 ps, and 5 ns) and wavelengths (1030 nm, 1064 nm, 532 nm). The damage probability curves and corresponding LIDT fluences are presented in Fig. 2. The beam polarization and crystal orientation are kept constants during all the tests. The uncertainty attached to the values of LIDT fluence is ~10% of the corresponding fluence value. It corresponds to the error bar related to the measurement of energy and beam size between the different experiments. For the sake of clarity, the error bars are not mentioned in the graphs. Sapphire samples prepared with optimal surface preparation (type A method) have higher resistance to laser damage when compared to samples prepared with type B method (see Fig. 2). This result is verified for all irradiation conditions of wavelength and pulse duration, even if it is much less marked in short laser – matter interaction regime (fs and ps) with respect to ns pulsed regime. The fluence transition between no damage (damage probability = 0) to systematic damage (damage probability = 1) ΔF is also more abrupt in the fs and ps damage tests confirming that damage is more deterministic in these irradiation conditions compared to the ns case.

**Figure 3:**
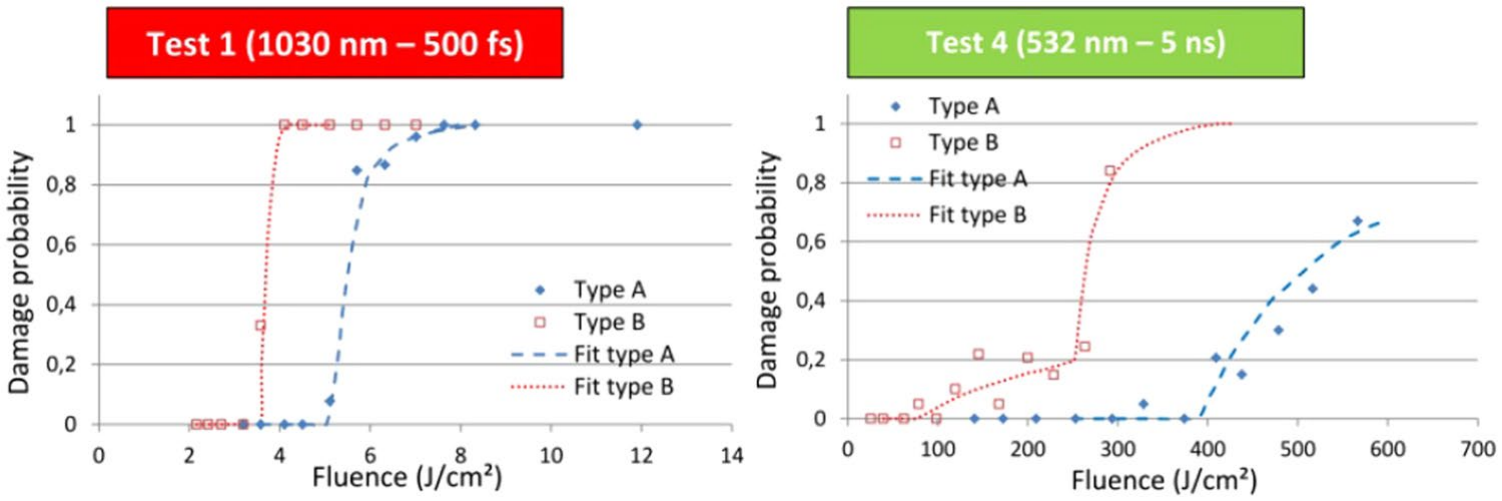
Density of defects. Damage probability curves and corresponding fits for tests 1 and 4 (as an illustration). The damage probability curves are fitted following a laser-induced damage model based on a two-parameter distributed defect ensemble. The values of defect densities retrieved for each test are summarized in Table 1.

### Correlation between PSD distribution of the samples and laser damage

**Figure 4:**
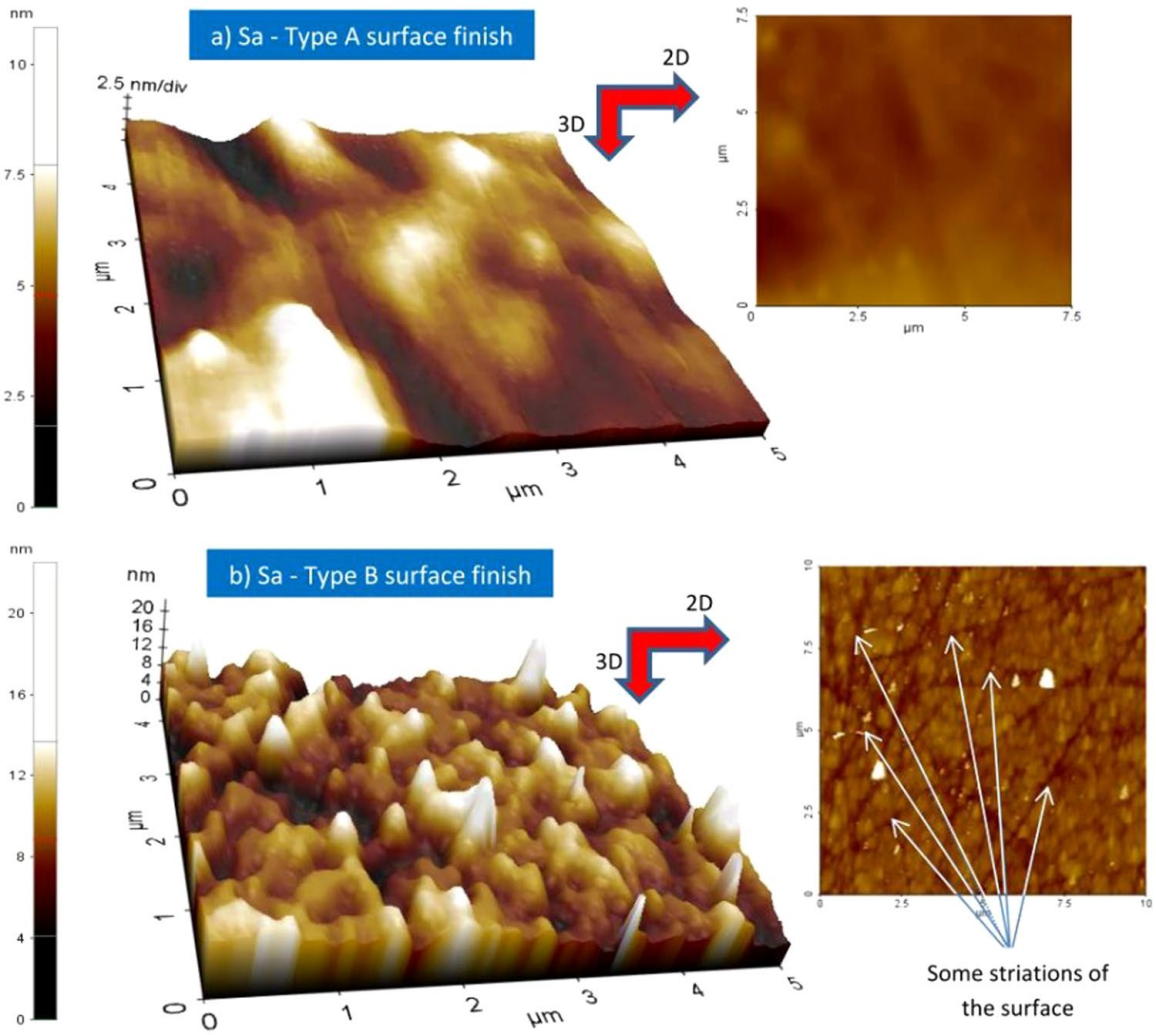
Striations and surface finish. 2D and 3D (zoomed in) surface topography of Sapphire samples of type A (**a**) and type B (**b**) surface finish. Note that the vertical scales are different. The white arrows indicate some striations of micrometric length and width ≪ λ (not exhaustive) that could be correlated with the reduction of LIDT in the case of type B surface finish (see text). The measured area roughly corresponds to a quarter of the typical surface illuminated during the laser tests.

Considering the surface data shown in Fig. 1 and even if the PSD distribution does not give a direct access to the spatial distribution of density of defects (it only yields the relative contribution of each spatial frequency to the surface texture)^28^, we can correlate the observed behavior with the high-frequency tail of the PSD distribution which is systematically measured when type B surface preparation is used. Indeed, for type A method, the surface topography is smooth and regular with only low-frequency PSD components, as shown in Fig. 1 and further in Fig. 4 and Table 1. The characteristic spatial lateral dimensions of main frequency components (Δλ_FWHM_ = 1.7–6.7 µm, see Fig. 1) are higher than the laser wavelengths used in the tests. These frequency components do not have any significant impact (enhancement) on the absorption of the laser energy at the surface of the material. On the contrary, for type B samples, the measured PSD distribution shows a large ΔF_FWHM_ frequency bandwidth. Indeed, it extends to high-frequency components with a characteristic cut-off wavelength (0.24 µm) smaller than the laser wavelengths used in the tests, which can be detrimental to damage resistance as we show hereafter.

**Table 1:** Surface density of defects used to fit the curves of Fig. 3.

Test	500 fs 1030 nm	50 ps 1064 nm	50 ps 532 nm	5 ns 532 nm
Type A surface finish Density of defects (mm^−2^)	30000	27000	10000	30000
Type B surface finish Density of defects (mm^−2^)	80000	80000	80000	2000/100000

### Importance of geometric topography defects on laser damage

The role of geometric defects of a surface (like cracks, grooves or striations, crevasses, etc.) on laser-induced damage was first studied by N. Bloembergen^29^, who showed that surface defects of width small compared to the laser wavelength induce a significant exaltation of the electric field. The local intensification of the electric field E is given by the formula:

1 $${\boldsymbol{E}}=\,\frac{2{{\boldsymbol{n}}}^{2}}{{{\boldsymbol{n}}}^{2}+1}{{\boldsymbol{E}}}_{0},$$

where E_0_ is the initial laser electric field and n the index of refraction of the material^29^. In the case of deep striations (with aspect ratio depth/diameter >1), the formula is simply: ${\boldsymbol{E}}={{\boldsymbol{n}}}^{2}{{\boldsymbol{E}}}_{0}$. More complex calculations of the laser electric field enhancement can be done when the exact morphology of the striations are known in details^30^. Note that in our case, the depth of the striations or crevasses is not known with high precision because of the difficulty to have at the same time a very good transverse and vertical spatial resolution with AFM when the surface is rough, especially when measuring striations or pores with aspect ratio superior to unity. However, taking into account the measurement of the roughness parameter Ra, we assume that the second case corresponding to deep striations is not encountered in our cases. For Sapphire (n = 1.7545 at 1064 nm) and considering that the index of refraction is a pure real (Sapphire being transparent at the wavelengths considered), the factor of over-intensification of the electric field is 1.5, which corresponds to a factor of 2.3 in intensity. Note that if considering the change in refractive index of the Sapphire with the wavelength (n = 1.7717 at 532 nm and n = 1.7545 at 1064 nm), this induces a variation of the intensification factor of ~1%. The ratio of LIDT fluences (F_th,typeA/_F_th,typeB_) between the two types of procedure of surface preparation is around 1.4–2 in fs and ps irradiation regimes and close to 5 for ns pulses. It stresses the correlation between the evolution of the resistance to laser damage of the material and the quality of surface finish and its resulting surface topography (in particular the topological defect of dimension ≪ λ as shown before). In fact, the activation of this kind of defect results from the local intensification of the electric field due to geometric aspect considerations. A significant dependence is expected on the laser wavelength because the occurrence of the field enhancement on such geometric aspect defect is related to the ratio of its characteristic lateral dimension to the laser wavelength. It can thus explain at least partly the important LIDT decrease observed for type B samples in ps regime (see Fig. 2) when the laser wavelength was varied from IR (1064 nm) to visible (532 nm). Combined with the higher sensitivity of laser absorption to defects observed in ns regime^26, 31^, it also explains the larger ratio of LIDT reduction (~5) that we have observed with ns pulse excitation at 532 nm during the damage tests.

As a first conclusion, it is interesting to note that the simple scaling provided by Eq. 1, correlated to the geometric characteristics of the surface finish put into evidence by our measurements, can explain reasonably the relative evolution of the damage thresholds of the samples prepared with the two procedures of surface finish.

### Retrieval of density of defects

In order to test the correlation of high-frequency PSD components with the LIDT reduction that we observed whatever the pulse duration on type B samples, we now fit the damage probability curves by a laser-induced damage model based on a two-parameter distributed defect ensemble. The principle and interest of this approach have been shown in several papers^32, 33^. The details of our modelling can be found elsewhere^34^. Briefly, the model assumes that the damage is initiated on defects or precursors randomly spread on the surface of the material and that any kind of defect is characterized by a local “activation” fluence (for a given laser, thus for a given pulse duration) to initiate damage. The model is based on Poisson law and Gaussian distribution to describe the damage probability and the activation fluence of a defect population. The number of defects per unit area (or density of defects) and activation fluence of the defect are used as free parameters to fit the experimental damage data. Depending on the cases, one or more class of defects (characterized by different densities and activation fluences) are retrieved from these calculations (see for instance the type B - ns case). Indeed, when the damage probability curve shows abrupt variations in the fluence transition range, this is interpreted as the presence of two (or more) populations of defects of different densities. This model only informs on the density of the defects present on the surface and not on their chemical nature or structure. It also does not yield any data on their characteristic geometric dimensions. However, it provides valuable qualitative information for understanding damage initiation in many cases. As an illustration of the procedure, we provide in Fig. 3 the curves corresponding to the fs and ns experiments and the ensemble of results issued from the fits are summarized in Table 1.

Considering the whole set of experiments and for a given procedure of surface preparation, the retrieved densities of defects are almost the same for all tests. When the samples are prepared following the type A procedure, a population of defects with a density of ~30000 mm^−2^ is put into evidence by the damage tests except for the case 50 ps – 532 nm for which a lower density is obtained. In the case of type B procedure for surface preparation, the characteristic density of defect is higher around 80000 mm^−2^. A second population of defects with a low density is also put into evidence in the case of type B surface preparation and under ns exposure. Not surprisingly, we also observe a large fluence transition range between no damage and systematic damage (ΔF > 200 J/cm²) in this temporal regime. It is in accordance with the higher sensitivity to defects and stochastic nature of laser damage observed with ns pulses^26, 31^.

## Discussion

Using the previous data, we establish a link between the density of defects and the high-frequency PSD components and further the reduction of the damage threshold observed with the samples prepared with type B method compared to those prepared with type A process. If we calculate from Table 1 the number of defects N_defect_ statistically present under the area illuminated by the lasers (we recall that w_0_ ~ 10 µm), we get N_defect,type B_ ~25. When observing the visual aspect of the surface, this is consistent with the number of striations of width ≪ λ and micrometric length which are scarcely widespread on the surface (see Fig. 4b). Thus, it seems to be reasonable to make the correlation between those striations and the high-frequency PSD tail, especially the cut-off frequency component which is present on the surface with a very low density as shown in the PSD measurement (see also Table 1).

However, it is not an absolute demonstration because i) the damage phenomenon is destructive and it can be multi-parameter and multi-scale and we cannot perform the damage test on a single isolated defect with infinite accuracy and ii) the PSD distribution only yields the relative contribution of each spatial frequency to the surface texture^28^. Finally, dedicated studies implying highly controlled structuration of the surface would be interesting to develop in the future to confirm this result and thus the exact characteristics of the striations responsible for the reduction of the laser damage threshold. Note also that the same analysis applied to type A sample yields a much lower value of defects with N_defect,type A_ ~9. However, we cannot do a similar correlation as the surface appears to be smooth and regular and free of any striations (in the limit of the capacity of detection of our surface characterization setup). In fact, the nature and characteristics of the defects that may be responsible for damage (and eventual LIDT limitation) in the case of type A samples cannot be determined from our experiments and analysis. They can be of extremely diverse origin, nature and characteristics, but nonetheless, the hypothesis of striations (like in case B) can be ruled out in the case of type A samples (high quality of surface finish). Moreover, if we further concentrate on the fs case (test 1), we observe that the transition ΔF is sharper when the surface finish is of lower quality (type B, ΔF_typeB_ ≅ ΔF_typeA_/3 ≅ 1 J/cm²). Note here that the amplitude of the damage transition ΔF is significantly higher than the shot-to-shot fluence fluctuations (estimated to be ~10% peak-to-valley) and to the fluence increment step used in the experiments making not ambiguous the existence of intermediate transition points in the damage probability curves. Not surprisingly, the LIDT fluence (type B) is also ~30% smaller compared to the surface preparation yielding higher surface quality (type A). In case of a pure dielectric material (like Sapphire crystal) exposed to the irradiation of a short pulse (especially considering femtosecond excitation), the laser energy absorption is nonlinear and takes place primarily on the electrons of the valence band promoting them to the conduction band by photo-ionization (multiphoton or tunnel ionization depending on the adiabaticity parameter)^35^. These seed electrons in the conduction band are further heated by Inverse Bremsstrahlung and multiplied by impact ionization finally leading to avalanche and optical breakdown of the dielectric material when the critical density is reached^24, 26^. In this case, and if one would intend to use the model presented before for interpreting the results, one should consider that all the electrons of the valence band are seen as the only potential seed (or precursor) of laser absorption in the material. As a result, it should translate into an extremely sharp fluence transition ΔF corresponding to a high density of defects close to the atomic density. However, to access such a level of information, it would also require a perfect idealized laser, without any fluctuations to provide always the same excitation, and infinitesimally small increment steps of energy (fluence) during the experiment to correctly evaluate the transition in the damage probability curve and get an accurate estimation of the density of these intrinsic “defects”. Such a defect model is thus not adapted to describe the situation of a pure dielectric material (without any extrinsic defect) exposed to femtosecond pulses. However, such a material corresponds to an idealized situation. Indeed, due to the process of fabrication or the structural arrangement of the atoms or molecules composing the lattice, any material will present a certain number of defects which can be of different kinds (structural, chemical, topological, etc). In addition, the surface supports in general a higher density of defects (as compared to the bulk) resulting from the surface making. Depending on their density and on their exact nature, in particular if they are able to trigger an absorption involving one or a low number of photons, they may influence the absorption (yielding to extrinsic absorption) of the laser energy and lower the laser-induced damage threshold fluence of the material. So the defect model is still interesting to apply even in the case of irradiation with femtosecond pulses. Because it may provide valuable information on the existence of some defects that may limit the damage threshold of a dielectric material and make it less resistant than its intrinsic limit, as it would be measured with a perfect dielectric material. This is indeed the purpose of the comparison of the damage curves obtained in femtosecond regime and showing differences, in terms of threshold, sharpness of damage transition ΔF and thus in terms of retrieved density of defects, between Sapphire prepared with the optimal procedure (type A, with a low density of defects) and the second procedure used classically for polishing Ti:Sapphire crystals (type B, with a higher density of defects). We can thus conclude that even in the femtosecond regime and with an optimal preparation of the surface of the material (type A), the damage LIDT fluence, which is characterized by a large damage transition (ΔF ≅ 3 J/cm²) and a significantly higher value (4.7 J/cm²) than the one measured with type B preparation (3.2 J/cm²), is still somewhat limited by the presence of low-density defects providing extrinsic channels of absorption. From our results and analysis, we cannot identify the origin of this limitation observed with type A surface preparation. However, we hypothesize that upon further optimization of the conditions of preparation of the material, we may continue to approach the highest (intrinsic) LIDT value that could be reached with a pristine material without any defects.

Moreover, the experiments in the fs regime have also revealed that type B surface preparation yields a low LIDT value and a sharp transition. This results from the presence of a population of high-density defect and acting as precursor sites for laser absorption. Interestingly, in this configuration, Sapphire could be advantageously used for laser ablation or surface modification because of i) the reduction of the necessary level of fluence required to modify the surface of the material and ii) the reduction of the ΔF transition (related to the higher density of defects) which in fact corresponds to the fluence range for which erratic damage (non-controlled modification) of the material is observed.

Finally, we compare the LIDT values measured at the surface with former results measured in the bulk on our damage test-bench in the same irradiation conditions^31^. Note that this comparison shall remain essentially qualitative because the volume of absorption and conditions of energy confinement and relaxation are significantly different at the surface and in the bulk. If we consider the LIDT values measured in the case of type B samples, we observe that they are much smaller (by a factor 2.1 to 3.2) than LIDT measured in the bulk^31^. For type A samples, the LIDT values are close to the bulk ones. Type A procedure for surface preparation thus appears to be a technique that does not significantly lower the resistance of the surface to laser damage. Therefore, it should be considered as close-to-optimal for the preparation of Sapphire crystals.

## Conclusions

We have compared the surface topography of Sapphire crystals resulting from two procedures of surface preparation used in the optics industry. Furthermore, we have studied their impact on surface damage of the crystals when they are exposed to single laser pulses and illumination conditions varied on three different pulse durations, from femtosecond to nanosecond, and on three laser wavelengths covering popular spectral laser emission lines in near-IR (1064 and 1030 nm) and visible (532 nm). Prior to performing the damage tests, the surface topography of Sapphire crystals was carefully characterized using an AFM system.

Our main results are the following:

1. *Relevance of developed methodology*. Considering the initial characterization of the surface of the samples, we note that the determination of the Power Spectral Density distribution (PSD) is more relevant for interpreting the observed damage behavior than the measurement of the average roughness parameter Ra. This is because the roughness parameter Ra only contains information on the height amplitude of hollows and bumps found on the surface but not on their spatial extent along the surface plane. More importantly, we define a methodology based on laser damage experiments and precise surface determination (in particular the importance of PSD determination) for fruitful analysis of the damage initiation at the surface of an optical material. This is the first demonstration of the high relevance of combining i) LIDT measurements, ii) AFM measurements and surface PSD determination, and iii) simple modeling to infer the corresponding density of defect, for providing detailed understanding of the surface damaging phenomenon and identifying the damage limitations of a material. Such methodology is in addition universal, can be applied to any material and will be helpful to deeply investigate physical mechanisms as complex as damage, or from an applicative viewpoint.
2. *Optimal surface preparation for Sapphire*. The procedure of surface preparation of Sapphire crystals shall provide a resultant surface topography smooth and regular to be not a limiting factor significantly lowering the resistance of the material to laser damage. In these close-to-optimal conditions (referred as type A surface finish in these works), high LIDT fluence can be accessed and the damage behavior of the material is presumably approaching its intrinsic properties. However, full verification of the high merit of this procedure of surface preparation would require the use of beams of large diameter (that cannot be provided by our damage set-up) for damage test and certification on the whole surface of the material.
3. *Evaluation of limitations of other surface preparation techniques*, *and route of improvement*. We have tested a second type of surface preparation (type B) which is used in particular for preparing the surface finish of Titanium-doped Sapphire crystals. We showed that this procedure of surface preparation lowers significantly the resistance to laser damage of Sapphire, by a factor ~1.4–2 in short pulse regime (ps and fs) and by a factor ~5 in long (ns) pulse regime. We explained the observed behavior by the presence of high-frequency modulations (with the characteristic dimension in the range of a fraction of the laser wavelength) and striations on the surface of the material. For Sapphire, we clearly demonstrate that this method of surface preparation or polishing is a factor limiting its resistance to laser damage. We also demonstrate that the weakness arises specifically from the high-frequency PSD components, enabling to provide precise recommendation to improve the procedure (suppression of high-frequency PSD components) in view of its application to the polishing of Titanium-doped Sapphire crystals, which is a crucial laser material in the context of the development of high-peak-power femtosecond laser systems towards unprecedented PW level^5^. This is even more important because we determined recently^31^ that such doped crystals have equal or even higher bulk-LIDT than pure Sapphire crystals whatever the investigated wavelengths (visible, near-IR) and pulse duration (fs to ns).

Moreover, as positive outcomes of the evaluation of the type B surface preparation, we also show that the macroscopic modification of type B Sapphire samples by femtosecond pulses is obtained at a small energetic level (low LIDT fluence) and with a high control of the laser-induced modification (ΔF_typeB_ ≪ ΔF_typeA_) as compared to Sapphire samples prepared with type A method. Those characteristics of processing are favorable in view of surface structuration of Sapphire, especially when the highest optical surface quality is not required by the application. This can be of interest for photonics and laser and optics industry since Sapphire is a material hard and delicate to machine.

## Materials and Methods

### Sapphire samples

We have performed tests on Sapphire crystals grown with the Czochralski technique and which surface has been prepared following two different procedures. As a general view, the process for surface preparation (polishing) is an abrasive technique that may yield chemical contamination, surface and sub-surface alterations or stress depending on the level of aggressiveness of the compounds and tools used^1, 9–12^. Surface alterations induced by the process of polishing may also provide local sites for attracting pollution or absorbing contaminants. Afterward, when the material surface is exposed to laser irradiation, the defects resulting from the process of surface preparation promote locally enhanced absorption of the laser field or reduce the resistance of the material to fracture^9, 13–16, 29, 30, 36^.

Considering our study, we did not get any information about the exact processing conditions used for the two methods of surface preparation and in these works, we refer them as “type A” and “type B”. The known points are that “type A” refers to the classical method of surface preparation of Sapphire windows used in optics industry and that “type B” corresponds to another technique employed for instance for supplying high-quality Titanium-doped Sapphire crystals for laser manufacturers. In fact, it is not possible to obtain the same quality of surface finish for doped and pure Sapphire crystals. The surface preparation of Sapphire crystals is usually performed by friction (fine grinding) using diamond beads, eventually combined with chemical actions to obtain the best final conditions of the surface^1^. In this process, the diameter of the beads determines the quality (surface roughness) of the resulting polishing. In the case of Sapphire crystals, one can use beads of small diameter to yield an optimum surface preparation (low surface roughness). However, when considering Titanium-doped Sapphire crystals, the same beads cannot be used because they make the surface much more brittle and fragile and thus unsuitable for further use in high-peak-power laser systems. The Ti:Sapphire crystals are thus prepared with diamond beads of larger diameter, yielding surfaces more rough as compared to Sapphire crystals. However, as the same technique of surface preparation is used, except the size of the beads, the respective surface finish differ mainly by the resulting geometric surface topography and not chemically that would be related to the use of different polishing materials or products and procedures.

### Surface characterization

In order to characterize the surface topography of the Sapphire samples, prepared with the two techniques of surface preparation, we used an AFM system (PSIA XE-100) before the damage tests. We measured the Ra roughness parameter which represents the average of the height difference (in the vertical direction normal to the sample surface) along with a specific distance with respect to a straight line parallel to the surface. In our characterization, the detection tool is the AFM tip and the scan distance is typically 10 µm, which corresponds approximately to the laser beam waist used in the damage tests. However, in the context of laser damage, the Ra parameter is not sufficient to describe the surface topography of a sample since it is not able to detect the isolated defects like striations or scratches of a surface and to provide a full description of the surface texture. The scratch and dig S/D parameter (as specified following the U.S. Military MIL-REF-13830B standard) is a classical data given by the optics supplier. In our case, the samples of type A and type B respectively have an S/D parameter of 10/5 and 40/20. These data provide extremely valuable information on the quality of the surface topography of an optical element and its subsequent use in an optical arrangement. Nevertheless, it remains qualitative since the S/D specification does not correspond to the measurement of the actual width of the scratch, but to the appearance of the scratch as compared to the standards^37^. Therefore, in order to provide a complete description of the surface topography of the sample, we measured its PSD with AFM. The PSD measurement specifies how each spatial frequency component contributes to the surface texture^28, 38^. In addition to the classical roughness parameter like Ra, the PSD data provide information on the lateral dimensions of the surface features and in particular on the modulations, striations and any irregularities which may be randomly observed on the sample surface as a result of the process of preparation of the surface.

### Laser damage tests

We used three different pulsed lasers (nanosecond (ns), picosecond (ps) and femtosecond (fs) sources) and similar damage set-ups in all experiments. The test bench has been presented elsewhere^31^ and we report only the details important for this study. The laser sources with nearly Gaussian beam distribution are linearly polarized and the energy is adjusted by a half-wave plate mounted on a motorized rotation stage coupled with a polarizer. We used focused laser spots with beam waist at 1/e² w_0_ = 10 µm ± 5 µm in all experiments. The relatively tight focusing allowed us to study damage of the front surface independently of the volume of the material. Indeed, the Rayleigh lengths z_R_ observed in the different tests are smaller than the thickness d of the studied crystals (z_R_ ≈ 225 µm–620 µm ≪ d, with d varying between 4 to 6 mm depending on the sample), allowing us to locate with high precision the beam waist at the surface of the samples with z-scan techniques.

We further irradiated the samples at different fluences (the energy E being the only varying parameter) and on different sites (typically 20) using the *1on1* procedure, i.e. using one laser pulse per site. The separation between two adjacent sites was sufficiently large (typically 5 times the beam diameter) to ensure a complete independence of each irradiation test. We obtained a damage matrix composed of n lines of 20 independent tests. Using *ex-situ* optical microscopy to detect the occurrence of laser damage, we finally plot the damage probability curves as a function of peak fluence (F = 2E/πw_0_²). The LIDT value corresponds to the highest fluence for which the damage probability is zero. This value indicates also the minimum level of energy density required to induce a macroscopic transformation of the surface of the material, while the fluence for which the damage probability is equal to one yields the energetic level to get a systematic modification of the surface of the material.
